# Impact of psychological resilience and social support on psycho-social adjustment in postoperative patients with primary hepatocellular carcinoma: mediating effects of fear of progression

**DOI:** 10.3389/fpsyg.2024.1461199

**Published:** 2024-10-08

**Authors:** Min Li, Binyang Yu, Haiyan He, Ning Li, Rui Gao

**Affiliations:** ^1^School of Nursing, Health Science Center, Xi 'an Jiaotong University, Xi 'an, China; ^2^Graduate School, Beijing University of Chinese Medicine, Beijing, China; ^3^Xiyuan Hospital, Chinese Academy of Chinese Medical Sciences, Beijing, China

**Keywords:** postoperative patients with primary hepatocellular carcinoma, psychological resilience, social support, fear of progression, psycho-social adjustment, mediating effects, structural equation model

## Abstract

**Background:**

Postoperative patients with primary hepatocellular carcinoma (HCC) confront not only physiological challenges but also psychological and social adaptation issues. It is imperative to enhance psycho-social adjustment (PSA) levels and further improve the quality of life among this population. However, research on PSA levels in postoperative HCC patients is lacking, and investigations into its associations with psychological resilience, social support, and fear of progression (FoP) remain unexplored currently.

**Objectives:**

This study aims to: (1) investigate the current status of PSA and analyze its influencing factors among postoperative HCC patients; (2) explore the interrelationships among psychological resilience, social support, FoP and PSA based on the Chronic Illness Adaptation Model employing a structural equation model.

**Methods:**

Convenience sampling methods were employed to recruit participants from the Department of Hepatobiliary Surgery at a tertiary hospital in Xi’an, Shaanxi, China, and a total of 399 patients completed the surveys. The survey instruments included a general information questionnaire, Connor-Davidson Resilience Scale (CD-RISC), Social Support Rating Scale (SSRS), Fear of Progression Questionnaire-Short Form (FoP-Q-SF), and Psychosocial Adjustment to Illness Scale Self-report (PAIS-SR). Data entry was conducted using Epidata 3.1 with dual verification, followed by statistical analyses performed using SPSS 27.0 and Amos 28.0.

**Results:**

The structural equation model revealed two paths. In Path 1 (psychological resilience → FoP → PSA), the direct effect was −0.383 (95% CI [−0.589, −0.112]), with an indirect effect of −0.075 (95% CI [−0.170, −0.018]). In Path 2 (social support → FoP → PSA), the direct effect was −0.297 (95% CI [−0.587, −0.063]), with an indirect effect of −0.069 (95% CI [−0.156, −0.019]).

**Conclusion:**

Postoperative patients with primary HCC exhibit lower levels of PSA. Higher levels of psychological resilience and social support correspond to elevated PSA levels. Conversely, advanced age, greater financial burden, and increased FoP are associated with lower PSA levels. FoP serves as a partial mediator between psychological resilience and PSA, as well as between social support and PSA. Future research would benefit from longitudinal designs to elucidate the developmental trajectories and causal links among these variables.

## 1 Introduction

### 1.1 Background

Primary liver cancer (PLC) ranks seventh globally in terms of incidence and second in mortality (Kulik and El-Serag, 2019). It predominantly comprises hepatocellular carcinoma (HCC), intrahepatic cholangiocarcinoma, and mixed hepatocellular-cholangiocarcinoma, with HCC alone constituting 75 to 85% of cases (Sung et al., 2021). Each year, approximately half of the new cases and deaths from PLC worldwide occur in China (Sung et al., 2021; Zheng et al., 2023). In 2022, China reported an estimated 367,700 new cases of PLC, with approximately 316,500 deaths attributed to the disease (Zheng et al., 2024). Primary HCC currently stands as the fourth most common malignant tumor and the second leading cause of cancer-related mortality in China, posing significant threats to public health (Cao et al., 2021).

The management of HCC necessitates a multidisciplinary approach, integrating various therapeutic modalities such as hepatic resection, liver transplantation, ablation therapy, transarterial chemoembolization (TACE), radiation therapy, and systemic anticancer treatments. Despite significant strides in HCC diagnosis and treatment in recent decades, there has been limited reduction in mortality rates. Approximately 70% of HCC cases in China are diagnosed at advanced stages, with notable peaks in postoperative recurrence observed at 6 months and 2 years, culminating in a five-year recurrence rate as high as 70% (Yang J.D. et al., 2019; Ding et al., 2021). Tumor recurrence, progression, metastasis, and liver failure are principal contributors to patient mortality during treatment. Postoperative patients encounter not only physiological challenges but also psychological and social adaptation issues.

Psycho-social adjustment (PSA) in cancer patients, also known as cancer adaptation, refers to the dynamic process during the post-diagnosis survival period where patients attempt to address emotional issues, resolve specific concerns related to cancer, and gain a sense of control over life events associated with the disease (Londono and McMillan, 2015). With the evolution of the bio-psychosocial medical model, integrating psychosocial aspects into the clinical care of malignancies has become an inevitable trend in medical development (Wu et al., 2015). Research indicates that the processes of surgery, chemotherapy, or radiotherapy for cancer can lead to significant changes in body image, such as hair loss, changes in body weight, alterations in skin condition, or physical disfigurement (Wu et al., 2023). These transformations can impact patients’ self-image and self-esteem, resulting in adverse alterations in self-perception (James, 2011). Concurrently, the treatment process can also induce dysfunction in the immune and endocrine systems, increasing the risk of infections and causing fluctuations in hormone levels, thereby affecting patients’ physiological functions and emotional states (Król et al., 2018; Hiam-Galvez et al., 2021). Furthermore, a decline in functional capabilities such as strength, endurance, and flexibility can restrict patients’ daily activities, further diminishing their sense of self-efficacy (Schover, 1991). Persistent pain and heightened self-focus on the body can exacerbate patients’ perception of internal discomfort, reducing their satisfaction and inducing anxiety (Sebri et al., 2023). Changes in social and interpersonal relationships can also affect their social identity and sense of self-worth (Rhondali et al., 2015). For HCC patients post-surgery, additional challenges include malnutrition caused by liver dysfunction (Wen et al., 2022), and severe complications such as infections, bleeding, and bile leaks (Forner et al., 2018). The use of targeted therapies and immunosuppressants can impair various body systems’ functionality (Serper et al., 2022; Vedadi et al., 2023), leading to a spectrum of symptom clusters related to gastrointestinal issues, fatigue, sleep, and neurocognitive impairments (Chen M. et al., 2024). These physiological, psychological, and social adaptation issues severely impact patients’ quality of life (Jiang et al., 2022; Tang, 2023).

It is noteworthy that employing effective coping strategies can assist cancer patients in better adapting to changes in physiological functioning, body image, social roles, and psychological states. Previous researches (Ahmadi et al., 2017; Sebri and Pravettoni, 2023) have demonstrated that Cognitive Behavioral Therapy (CBT), tailored psychological interventions, and group-based body compassion interventions can significantly improve body image impairments in breast cancer patients and women with infertility, reduce fear of disease, and foster the reconstruction of social relationships. However, there is currently a lack of effective psychological interventions for the series of psychological and social adaptation issues faced by postoperative patients with primary HCC. Psychological resilience acts as an internal coping mechanism. Numerous studies have indicated that individuals with higher levels of psychological resilience achieve more favorable clinical outcomes, including a reduction in symptoms, decreased anxiety and depression, accelerated disease recovery, enhanced self-efficacy, and an improved quality of life (Lee et al., 2019; George et al., 2023). Social support, as an external coping resource, from various sources, facilitates positive reassessment of one’s circumstances, promotes social cognitive changes, enhances problem-solving abilities, and strengthens stress coping mechanisms, effectively mitigating negative stress-related impacts. Good social support aids in faster recovery of patients’ physical and mental health, facilitating the realization of their self-worth (Ma et al., 2013).

It should be emphasized that fear of progression (FoP) is a prevalent psychological state among cancer patients. Studies indicate that approximately 38 to 97% of cancer patients experience varying degrees of FoP, with about 49% of cancer survivors experiencing moderate to severe levels (Koch et al., 2013; Vandraas et al., 2021). Excessive FoP can escalate into a pathological state, increasing symptom burden and leading to serious mental illnesses such as somatic symptom disorders or post-traumatic stress disorder (Crist and Grunfeld, 2013; Tsai et al., 2018; Yang Y. et al., 2019). This can manifest in negative behavioral changes (e.g., avoidance of monitoring, medication refusal, repeated cancellations or delays of follow-up examinations) and unhealthy lifestyle habits (e.g., smoking, alcohol consumption, decreased adherence to physical exercise and healthy diet) (Thewes et al., 2012; Shim et al., 2020), severely impacting disease management, prognosis, and patient quality of life. Study indicates a negative correlation between fear of cancer recurrence and PSA levels (Niu, 2023).

In summary, considering the high incidence of HCC in China and the significant rate of postoperative recurrence, along with the pervasive and intense FoP among cancer patients, there is an urgent need to address FoP following surgery and to enhance PSA, thereby improving patients’ psychological well-being and quality of life. However, research on PSA levels in postoperative patients with primary HCC is currently scarce, and the associations between PSA, psychological resilience, social support, and FoP have yet to be explored in the existing literature.

### 1.2 Objectives

Therefore, this study aims to: (1) investigate the current status of PSA and analyze its influencing factors among postoperative HCC patients; (2) explore the interrelationships among psychological resilience, social support, FoP and PSA based on the Chronic Illness Adaptation Model by constructing a structural equation model; (3) further provide clear directions for developing personalized and targeted psychological interventions to enhance patients’ mental health and to guide clinical nursing practices and health management policies.

## 2 Materials and methods

### 2.1 Participants and procedure

This study employed a non-random, convenience sampling method. From January 2024 to May 2024, postoperative patients with primary HCC were recruited from the hepatobiliary surgery wards and outpatient clinics of a tertiary hospital in Xi’an, Shaanxi, China. Both on-site and online questionnaire surveys were conducted. Prior to questionnaire distribution, investigators were uniformly trained to ensure comprehension of all items’ content and objectives, and to use standardized instructions for informing patients about the requirements for completing the survey. Participants were selected strictly according to the inclusion and exclusion criteria, resulting in a total collection of 410 cases. Inclusion criteria were as follows: (1) aged 18 years or older; (2) diagnosed with primary HCC based on WHO diagnostic criteria and pathological examination, and had undergone surgery (including liver resection, liver transplantation, or interventional surgery); (3) capable of reading and understanding Chinese-language questionnaires and communicating verbally; (4) willing to participate and sign informed consent forms. Exclusion criteria included: (1) patients with metastatic liver cancer or other non-HCC types of liver cancer; (2) individuals with other severe diseases, such as severe cardiopulmonary disorders, renal insufficiency, or severe mental illnesses; (3) patients with cognitive impairments or altered consciousness who are unable to comprehend the research procedures. Inpatients were administered and collected questionnaires through face-to-face encounters. After completion, researchers immediately retrieved and checked each questionnaire on a item-by-item basis, inquiring and filling in any missing information. The survey took approximately 20–30 min to complete. For elderly patients and those with lower levels of education, researchers provided objective, non-leading explanations of the questionnaire items to assist them in completing the survey. Outpatients were surveyed using WeChat’s online platform, “Questionnaire Star,” which included reverse items to prevent forwarding, ensuring the quality of the questionnaire. Collected surveys were numbered and recorded, and invalid questionnaires were discarded. A total of 399 valid questionnaires were obtained, with a valid response rate of 97.3%.

This study received review and approval from the Institutional Review Board (IRB) of our institution, with ethics approval number (XJTU1AF2024LSYY-125). Throughout the study period, strict adherence to the principles of informed consent, confidentiality, and non-harm was maintained.

### 2.2 Survey tools

#### 2.2.1 General information questionnaire

We used a self-designed general information questionnaire by the research team to investigate the demographic and disease-related characteristics of postoperative patients with primary HCC, mainly including 9 demographic data such as age, gender, marital status, education level, etc., as well as 7 pathological data such as disease course, surgical time, liver cancer staging, surgical method, and Child-Pugh classification of liver function, etc., totaling 16 items.

#### 2.2.2 Connor-Davidson Resilience Scale (CD-RISC)

The Connor-Davidson Resilience Scale (CD-RISC) was used to assess the level of psychological resilience among patients postoperatively treated for primary HCC. Developed by Connor and Davidson in 2003 (Yu and Zhang, 2007), this scale measures individuals’ capacity to adapt positively to adverse conditions. It consists of three dimensions: resilience (13 items), strength (8 items), and optimism (4 items), totaling 25 items. Each item is rated on a 5-point Likert scale ranging from 0 (never) to 4 (always). The total scores range from 0 to 100, with higher scores indicating higher levels of psychological resilience. In this study, the overall Cronbach’s *α* coefficient for the scale was 0.974.

#### 2.2.3 Social Support Rating Scale (SSRS)

The Social Support Rating Scale (SSRS) was employed to evaluate the level of social support among patients postoperatively treated for primary HCC (Zou et al., 2023). The scale comprises 10 items across 3 dimensions: subjective support (items 1, 3, 4, 5), objective support (items 2, 6, 7), and utilization of support (items 8, 9, 10). Items 6 and 7 are scored based on multiple selections, where responding “none” scores 0 points, and selecting “the following sources” scores points corresponding to the number of sources chosen. Other items are scored individually based on their respective options. The total score ranges from 0 to 66, with scores ≤22 indicating low, 23 to 44 indicating moderate, and 45 to 66 indicating high levels of social support. In this study, the overall Cronbach’s *α* coefficient for the scale was 0.730.

#### 2.2.4 Fear of Progression Questionnaire-Short Form (FoP-Q-SF)

The Fear of Progression Questionnaire-Short Form (FoP-Q-SF) was utilized to assess patients’ levels of fear of disease progression. Developed by Mehnert et al. (2006) in 2006, this questionnaire was simplified from the Fear of Progression Questionnaire. The Chinese version of the scale was translated and validated by domestic researchers including Wu et al. (2015) in 2015, specifically for assessing the severity of fear of disease progression among liver cancer patients. The scale consists of two dimensions: physiological health (6 items) and family/social (6 items), totaling 12 items. Responses are rated on a 5-point Likert scale (1–5), with total scores ranging from 12 to 60. Higher scores indicate greater levels of FoP. In this study, the overall Cronbach’s *α* coefficient for the scale was 0.903.

#### 2.2.5 Psychosocial Adjustment to Illness Scale Self-report (PAIS-SR)

The Psychosocial Adjustment to Illness Scale Self-report (PAIS-SR) was employed to assess the PSA levels of postoperative patients with primary HCC. Constructed by Derogatis (1986) and translated into Chinese by Yao et al. (2013), this scale evaluates patients’ psychological and social adaptation following illness. It comprises 44 items across 7 dimensions: health care, work capacity, family relationship, sexual ability, communication, leisure activity, and psychological status. Using a 4-point Likert scale (0–3 points per item), total scores range from 0 to 132, where higher scores indicate lower levels of PSA. Scores ≥51 are classified as low adjustment, 35–51 as moderate adjustment, and ≤ 35 as high adjustment levels. In this study, the overall Cronbach’s α coefficient for the scale was 0.939.

### 2.3 Data analysis

Initially, data entry was conducted using Epidata 3.1 with dual verification. Subsequently, statistical analyses were performed using SPSS 27.0 and Amos 28.0. Descriptive statistics and factor analysis were conducted using SPSS. Frequency and composition ratios were used to describe categorical data. For continuous data, whether normally distributed or not, means ± standard deviations or medians (interquartile ranges) were reported accordingly. Parametric tests such as t-tests and one-way ANOVA were employed for normally distributed and homoscedastic data, while non-parametric tests were used for non-normally distributed data. Pearson correlation analysis was used to examine relationships between variables. Variables showing statistical differences in the univariate analyses were included in multivariate linear regression models. A structural equation model encompassing psychological resilience, social support, FoP, and PSA was constructed using Amos 28.0. Model fit was assessed using fit indices including χ^2^/*df*, Tucker Lewis Index (TLI), Comparative Fit Index (CFI), Incremental Fit Index (IFI), Goodness of Fit Index (GFI), Adjusted GFI (AGFI), and Root Mean Squared Error of Approximation (RMSEA) (Wen et al., 2004). Adequate model fit was indicated by χ^2^/*df* between 1 and 3, and values exceeding 0.9 for TLI, CFI, IFI, GFI, and AGFI, with RMSEA less than 0.08. Bollen-Stine bootstrap method was employed for further model refinement. Finally, bias-corrected non-parametric percentile bootstrap method was used to examine direct and indirect effects among variables. Confounding factors significantly associated with outcome variables in the multivariate analysis were controlled for, and changes in path coefficients and effect values before and after controlling for confounders were compared.

## 3 Results

### 3.1 Participants’ characteristics

Among 399 postoperative patients with primary HCC, the majority were males (298, accounting for 74.70%); The age range was between 25 and 85 years old, with an average age of 55.62 ± 11.90 years old; 96.00% of patients were married; In terms of educational level, the highest proportion was college graduates or above (142 people, accounting for 35.60%); A large proportion of patients reported no religious affiliation (96.50%); In terms of occupation, farmers had the highest number (155 people, accounting for 38.8%), followed by employed individuals (28.6%), retirees (22.6%), and unemployed (10%); Nearly half of the patients reported heavy (48.90%) or moderate (50.10%) financial burdens. Regarding disease-related data, the majority of patients (48.10%) had a disease duration of less than 6 months; Most patients were diagnosed at stage III (40.10%) or stage I (38.10%) of HCC. The majority had Child-Pugh class A liver function (69.40%). The most common surgical treatment was interventional therapy (49.40%), followed by hepatectomy (31.60%), with liver transplantation being the least common (19.00%). A majority of patients (63.90%) did not have any other chronic diseases, while 68.90% had a history of hepatitis. There were statistically significant differences in the scores of PSA levels among patients with primary HCC after surgery, including gender, age, marital status, education level, home location, religious beliefs, occupation, monthly income level, financial burden, surgical method, postoperative time, and immunosuppressive agent use (*p* < 0.05). Detailed information is provided in Table 1.

**Table 1:** Characteristics of participants and difference in PSA (*n* = 399).

Variables	Category	*n* (%)	Mean ± SD	*t*/*F*	*p*
Gender	Male	298 (74.7)	56.89 ± 16.12	−4.446^a^	<0.001
	Female	101 (25.3)	65.29 ± 16.92		
Age	25–44 years old	73 (18.30)	57.25 ± 16.95	11.212^b^	<0.001
	45–64 years old	221 (55.40)	56.54 ± 16.83		
	≥65 years old	105 (26.30)	65.47 ± 14.59		
Marital status	Married	383 (96.00)	58.66 ± 16.58	−2.128^a^	0.034
	Unmarried/divorced/widowed	16 (4.00)	67.69 ± 17.89		
Educational level	College degree or above	142 (35.60)	53.25 ± 17.19	15.573^b^	<0.001
	High / vocational school	91 (22.80)	57.97 ± 14.28		
	Middle school	96 (24.10)	61.64 ± 16.75		
	Primary school and below	70 (17.50)	68.49 ± 13.58		
Home location	Town	214 (53.60)	54.26 ± 16.78	−6.344^a^	<0.001
	Rural area	185 (46.40)	64.43 ± 14.94		
Religious belief	Yes	14 (3.50)	50.21 ± 23.21	−2.015^a^	0.045
	No	385 (96.50)	59.34 ± 16.38		
Occupation	Unemployed	40 (10.00)	57.03 ± 14.96	11.407^b^	<0.001
	Farmer	155 (38.80)	64.81 ± 15.61		
	Employed	114 (28.60)	54.11 ± 17.80		
	Retirees	90 (22.60)	56.14 ± 15.06		
Monthly income	<3,000	146 (36.60)	62.95 ± 17.16	6.897^b^	<0.001
	3,000 ~ 6,000	164 (41.10)	58.60 ± 14.95		
	6,000 ~ 10,000	83 (20.80)	53.89 ± 17.58		
	>10,000	6 (1.50)	45.67 ± 14.72		
Financial burden	Relatively light	4 (1.00)	22.50 ± 15.46	19.485^b^	<0.001
	Moderate	200 (50.10)	56.03 ± 15.81		
	Heavy	195 (48.90)	62.83 ± 16.17		
Disease course	<6 months	192 (48.10)	60.28 ± 16.00	0.969^b^	0.407
	6 ~ 12 months	53 (13.30)	59.45 ± 16.60		
	1 ~ 3 years	65 (16.30)	57.86 ± 17.18		
	Over 3 years	89 (22.30)	56.88 ± 17.90		
Liver cancer staging	I stage	152 (38.10)	58.48 ± 16.30	1.703^b^	0.166
	II stage	57 (14.30)	59.46 ± 16.44		
	III stage	160 (40.10)	60.47 ± 16.76		
	IV stage	30 (7.50)	53.17 ± 18.37		
Child-Pugh classification of liver function	A grade	277 (69.40)	59.57 ± 15.81	2.210^b^	0.111
	B grade	104 (26.10)	58.92 ± 18.18		
	C grade	18 (4.50)	51.06 ± 20.14		
Surgical treatment	Hepatectomy	126 (31.60)	58.71 ± 15.85	6.357^b^	0.002
	Liver transplantation	76 (19.00)	53.45 ± 20.03		
	Interventional therapy	197 (49.40)	61.37 ± 15.34		
Postoperative time	<6 months	267 (66.90)	60.26 ± 16.04	5.074^b^	0.007
	6 months to 1 year	62 (15.50)	60.13 ± 16.73		
	>1 year	70 (17.50)	53.30 ± 18.19		
Using targeted drugs	Yes	105 (26.30)	61.11 ± 14.06	1.500^a^	0.134
	No	294 (73.70)	58.27 ± 17.52		
Using immunosuppressants	Yes	75 (18.80)	53.08 ± 20.02	−3.462^a^	<0.001
	No	324 (81.20)	60.39 ± 15.56		
Other chronic diseases	Yes	144 (36.10)	59.74 ± 16.28	0.645^a^	0.519
	No	255 (63.90)	58.61 ± 16.97		
Hepatitis history	Yes	275 (68.90)	59.19 ± 16.58	0.305^a^	0.760
	No	124 (31.10)	58.64 ± 17.06		

^at value; bF value.^

### 3.2 Descriptive statistics and bivariate correlations among variances

The total scores of psychological resilience, social support, FoP, and PSA in postoperative patients with primary HCC were 52.79 ± 15.99, 39.04 ± 5.55, 35.18 ± 6.26, and 59.02 ± 16.71, respectively. These scores indicate levels that are moderate to low. Correlation analysis showed that there was a pairwise correlation (*p* < 0.05) between the total score of psychological resilience, social support, FoP, and PSA. The total score of social support was significantly positively correlated with scores in psychological resilience (*p* < 0.05), and negatively correlated with scores in FoP and PSA (*p* < 0.05). There was a significant negative correlation (*p* < 0.05) between the total score of psychological resilience and scores in FoP and PSA. There was a significant positive correlation (*p* < 0.05) between scores in FoP and PSA. Detailed information is provided in Table 2.

**Table 2:** Mean (M), standard deviations (SD), and correlations between the variables (*n* = 399).

Variables	Mean	SD	Psychological resilience	Social support	FoP	PSA
Psychological resilience	52.79	15.99	1			
Social support	39.04	5.55	0.538***	1		
FoP	35.18	6.26	−0.469***	−0.380***	1	
PSA	59.02	16.71	−0.667***	−0.531***	0.501***	1

***p<0.001.

### 3.3 Multiple regression analysis

Based on the analysis results, five variables entered the multiple linear regression equation with statistical significance (*F* = 27.195, *p*<0.001). The equation’s coefficient of determination was *R*^2^ = 0.577, adjusted *R*^2^ = 0.556, indicating that these five variables collectively explain 55.6% of the variance in PSA. In order of contribution, these variables were psychological resilience (*β* = −0.456), FoP (*β* = 0.233), financial burden (*β* = 0.193), age (*β* = 0.189), and social support (*β* = −0.159). Higher levels of psychological resilience and social support were associated with lower scores in PSA, indicating higher PSA levels. While older age, greater financial burden, and higher FoP were associated with higher scores in PSA, indicating lower PSA levels (refer to Table 3 for details).

**Table 3:** Results of multiple linear regression analysis (*n* = 399).

Variables	*B*	SE	*β*	*t*	*p*	VIF
Constant	33.347	16.135		2.067	0.039	
Age	0.265	0.064	0.189	4.138	<0.001	1.863
Financial burden	6.216	1.501	0.193	4.142	<0.001	1.953
Psychological resilience	−0.477	0.051	−0.456	−9.426	<0.001	2.099
Social support	−0.480	0.138	−0.159	−3.479	0.001	1.877
FoP	0.621	0.117	0.233	5.308	<0.001	1.724

### 3.4 Model goodness of fit test

**Figure 1:**
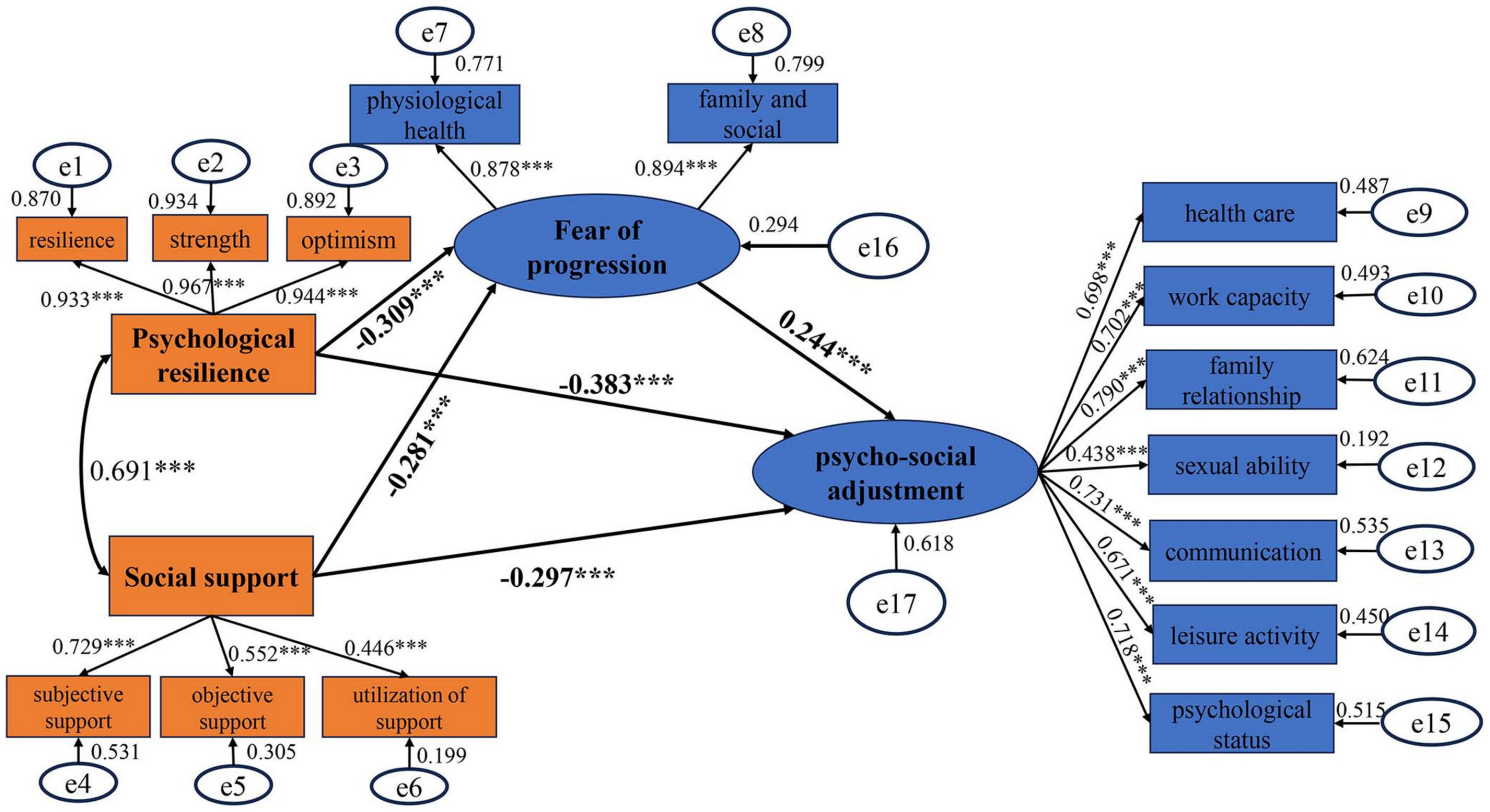
Pathway model diagram of psychological resilience, social support, fear of progression, and psycho-social adjustment.

The results showed that the structural equation model of the relationship between psychological resilience, social support, FoP, and PSA had good fit with the sample data. The model adaptation index was χ^2^/*df* = 1.403, RMSEA = 0.032, and TLI, CFI, IFI, GFI, and AGFI were all greater than 0.9. The model is shown in Figure 1.

### 3.5 Control and testing for common method bias

The survey tools used in this study were all based on self-report questionnaires, which may introduce common method bias. We employed the Harman single-factor test, with criteria that more than one factor should have eigenvalues greater than 1, and the largest factor should explain less than 40% of the variance (Xiong et al., 2012). Our results indicated that 17 factors had eigenvalues greater than 1, with the variance explained by the first factor at 27.77%, below the critical threshold of 40%. Therefore, there was no significant common method bias detected in this study.

### 3.6 Test for direct effects

The results indicated the presence of five direct pathways among the four variables: psychological resilience, social support, FoP, and PSA. Unstandardized coefficients primarily assessed the significance of results, while standardized coefficients evaluated the significance of each path coefficient. All unstandardized coefficients in the constructed model were significant. Both psychological resilience and social support exhibited direct negative effects on scores in PSA (*β* = −0.383, *p*<0.001; *β* = −0.297, *p* = 0.001). Additionally, psychological resilience and social support demonstrated direct negative effects on FoP (*β* = −0.309, *p*<0.001; *β* = −0.281, *p* = 0.006), whereas FoP had a direct positive effect on scores in PSA (*β* = 0.244, *p*<0.001). Therefore, psychological resilience and social support may indirectly influence PSA through FoP. Further controlling for confounding factors such as age and financial burden in multiple linear regression, the results showed that the coefficients of each pathway remained largely unchanged (see Figure 1 and Table 4 for detailed information).

**Table 4:** Results of pathway analysis.

Direct pathway	Non-standardized path coefficient	Standard error	C.R.	*p*	Standardized path coefficient
No controlling for confounding factors					
Psychological resilience→FoP	−0.112	0.032	−3.472	<0.001	−0.309
Psychological resilience→PSA	−0.097	0.019	−5.167	<0.001	−0.383
Social support→FoP	−1.044	0.38	−2.749	0.006	−0.281
Social support→PSA	−0.776	0.237	−3.272	0.001	−0.297
FoP → PSA	0.172	0.039	4.434	<0.001	0.244
Controlling for confounding factors					
Psychological resilience→FoP	−0.115	0.032	−3.619	<0.001	−0.319
Psychological resilience→PSA	−0.096	0.018	−5.371	<0.001	−0.388
Social support→FoP	−1.016	0.384	−2.648	0.008	−0.267
Social support→PSA	−0.740	0.235	−3.141	0.002	−0.284
FoP → PSA	0.158	0.04	3.992	<0.001	0.232

### 3.7 Test for the mediating effects

Based on Bootstrap mediation analysis standards (Fang and Zhang, 2012), if the 95% confidence interval (CI) for the indirect effect does not include 0, the mediation effect is considered significant; if it includes 0, the mediation effect is considered non-existent. If the 95% CI for the direct effect does not include 0, it indicates that the variable partially mediates the effect; if it includes 0, it suggests the variable fully mediates the effect. The study results indicated that FoP partially mediates between psychological resilience and PSA in patients after primary HCC surgery, as well as between social support and PSA. Before controlling for confounding factors, in Path 1: psychological resilience →FoP → PSA, the direct effect of psychological resilience on PSA was −0.383, with a Bootstrap 95% CI of (−0.589, −0.112), and the indirect effect was −0.075, with a Bootstrap 95% CI of (−0.170, −0.018), both excluding 0. The total effect was −0.459, with direct and indirect effects constituting 83.4 and 16.6% of the total effect, respectively. In Path 2: social support → FoP → PSA, the direct effect of social support on PSA was −0.297, with a Bootstrap 95% CI of (−0.587, −0.063), and the indirect effect was −0.069, with a Bootstrap 95% CI of (−0.156, −0.019), both excluding 0. The total effect was −0.365, with direct and indirect effects constituting 84.0 and 16.0% of the total effect, respectively. After controlling for confounding factors such as age and financial burden, the effects remained largely unchanged (see Table 5 for further details).

**Table 5:** Analysis of the effects of mediating models.

Pathway	Effects	Effect value	Standard error	95%CI		*p*	Percentage of total effect (%)
				Lower	Upper		
No controlling for confounding factors							
Psychological resilience→FoP → PSA	Direct effect	−0.383	0.120	−0.589	−0.112	0.010	83.4
	Indirect effect	−0.075	0.038	−0.170	−0.018	0.006	16.6
	Total effect	−0.459	0.118	−0.655	−0.191	0.007	–
Social support→FoP → PSA	Direct effect	−0.297	0.136	−0.587	−0.063	0.011	81.3
	Indirect effect	−0.069	0.034	−0.156	−0.019	0.010	18.7
	Total effect	−0.365	0.133	−0.652	−0.131	0.002	–
Controlling for confounding factors							
Psychological resilience→FoP → PSA	Direct effect	−0.388	0.111	−0.579	−0.137	0.007	84.0
	Indirect effect	−0.074	0.038	−0.168	−0.015	0.009	16.0
	Total effect	−0.462	0.107	−0.639	−0.215	0.003	–
Social support→FoP → PSA	Direct effect	−0.284	0.127	−0.549	−0.052	0.013	82.1
	Indirect effect	−0.062	0.034	−0.154	−0.014	0.014	17.9
	Total effect	−0.346	0.123	−0.615	−0.124	0.002	-

## 4 Discussion

To the best of our knowledge, our study is the first to explore the current status of PSA among patients after surgery for primary HCC, the influencing factors, and the relationships with psychological resilience, social support, and FoP. The findings indicate that PSA is at a relatively low level among these patients. Through correlation analysis and multiple linear regression analysis, we identified psychological resilience, social support, FoP, age, and financial burden as independent influencing factors of PSA. Higher levels of psychological resilience and social support are associated with lower levels of FoP and better PSA. Older age and greater financial burden are associated with lower levels of PSA. By constructing a structural equation model, we found that FoP plays a partial mediating role in the impact of psychological resilience and social support on PSA. Our research emphasizes that enhancing psychological resilience and social support for patients after primary HCC surgery can reduce the level of FoP, thereby improving their PSA and ultimately promoting better mental health and quality of life.

In this study, the overall level of PSA among postoperative patients with primary HCC is low. Multiple studies (Niu, 2023; Pan et al., 2023; Zhao, 2024) have indicated that patients who have undergone surgery for breast cancer exhibit moderate to low levels of PSA, and similar findings have been reported for patients post-surgery for colorectal cancer (Pang, 2023). Additionally, patients undergoing chemotherapy for lung cancer have been found to have moderate levels of PSA (Cui, 2023). These results are congruent with the conclusions drawn from our research, collectively underscoring the suboptimal PSA capabilities among cancer patients following surgical interventions. Cancer patients often face various challenges during treatment and recovery, including mental stress, emotional fluctuations, and adjustments in social roles, which contribute to decreased PSA (Omne-Pontén et al., 1992; Mader et al., 2022). For postoperative patients with primary HCC, the physiological recovery process post-surgery may involve bodily pain, fatigue, and lifestyle limitations, all of which can negatively impact patients’ mental well-being. Moreover, HCC, as a significant illness, introduces uncertainties in treatment, unclear treatment outcomes, and potential risks of recurrence, leading to fear and anxiety about the future, thereby affecting their PSA. Notably, the findings of this study indicate that postoperative patients with primary HCC exhibit moderate to low levels of psychological resilience and social support. Deficiencies in social support systems, constraints on healthcare resources, and variations in individual psychological resilience are also significant factors contributing to reduced levels of PSA (Martz et al., 2005; Chen et al., 2022).

Patients exhibiting higher levels of psychological resilience demonstrate superior PSA. Psychological resilience refers to an individual’s ability to adjust in the face of challenges, stress, and adversity, aiding patients in coping with psychological fears and uncertainties related to disease progression (Troy et al., 2023). For postoperative patients with primary HCC, the surgical and treatment processes present both physiological and psychological challenges, compounded by disease uncertainty and treatment side effects. Patients with higher psychological resilience may adopt more proactive psychological coping strategies, better manage the uncertainty of HCC progression and treatment, reduce FoP, and subsequently enhance their PSA. Studies (Chen S. et al., 2024; Duran et al., 2024; Mollaei et al., 2024; Yang and Liu, 2024) have also indicated positive associations between psychological resilience and the mental health, quality of life, and coping strategies of patients with breast cancer, prostate cancer, and colorectal cancer. These studies indirectly support the conclusions drawn from our research, collectively emphasizing the pivotal role of psychological resilience in the postoperative PSA of cancer patients.

Social support is equally crucial for the PSA of postoperative patients with primary HCC. It encompasses emotional support (such as comfort, understanding, and caring) and cognitive support (including information provision, advice, and feedback) (Zhang et al., 2024), which can originate from family, friends, and medical teams, providing patients with understanding, support, and relief from the psychological burdens of illness, thereby enhancing PSA (Ma et al., 2024). Both domestic and international researches indicate that strong social support can reduce anxiety and depression levels among cancer patients, promoting their psychological well-being (Chirico et al., 2024; Fan et al., 2024; Fu et al., 2024; Ma et al., 2024). Multiple domestic studies (Deng et al., 2019; Cheng, 2021; Zhou et al., 2022) focusing on patients after primary HCC surgery have identified social support as a significant factor in reducing patients’ FoP. For postoperative patients with primary HCC, receiving social support can alleviate psychological stress and feelings of loneliness during the recovery process, bolstering their sense of psychological security and confidence in coping with the illness and treatment. This support helps patients approach the challenges of recovery more proactively, thus maintaining their adaptive capacities across various dimensions (Hu et al., 2023).

Conversely, elevated levels of FoP are correlated with diminished PSA among postoperative patients with primary HCC. This finding aligns with research in breast cancer (Niu, 2023) and thyroid cancer (Ma, 2022), indicating that individuals with heightened concerns regarding their disease and its uncertainties exhibit diminished adaptive capacities in psychological and social realms. In our study, concerning patients who have undergone surgery for primary HCC, their FoP encompasses excessive fears and anxieties related to physical health and social and familial aspects. This psychological trepidation can exacerbate physical discomfort, influence adherence to treatment, work performance, social activities, and leisure in daily life. Meanwhile, PSA encompasses dimensions such as healthcare utilization, work capacity, and familial relationships. Consequently, an excessive level of FoP directly leads to a decline in PSA (Xiong et al., 2024).

Age and financial burden are also independent influencing factors of PSA. Older age is correlated to poorer PSA, consistent with findings from the study (Celik and Ozkan, 2018). Firstly, advancing age is associated with a gradual decline in physiological function and immune system efficacy among the elderly, rendering them more susceptible to the impacts of illness, including recovery and maintenance of life quality post-surgery (Pang, 2023). Secondly, older patients may experience significant changes in social roles and identities post-surgery, such as retirement and offspring becoming independent. These changes can lead to psychological maladjustment, impacting their performance in family relationships and communication. Furthermore, advancing age is accompanied by diminished psychological resilience, making elderly patients more prone to feelings of helplessness, anxiety, or depression when confronting the challenges of illness. Participation in recreational activities tends to decrease significantly among elderly patients post-surgery due to various discomforts, all of which can adversely affect their PSA. Heavier financial burdens are associated with poorer PSA, consistent with findings in studies (Lee and Kim, 2024; Zhou et al., 2024). The substantial financial strain can lead to anxiety, depression, and other negative emotions, impairing normal functioning in areas such as work capacity and family relationships, thereby affecting their psychological state and level of social adaptation.

Notably, our research findings indicate that FoP plays a partial mediating role between psychological resilience and PSA in patients following primary HCC surgery, as well as between social support and PSA. The pathway for psychological resilience is as follows: psychological resilience → FoP → PSA, with direct and indirect effects accounting for 83.4 and 16.6% of the total effect, respectively. Psychological resilience has a direct positive impact on PSA, while simultaneously exerting a direct negative influence on FoP, while FoP negatively affects PSA. Consequently, patients with higher levels of psychological resilience may mitigate FoP, thereby reducing its adverse impact on PSA. For social support, the pathway is social support → FoP → PSA, with direct and indirect effects constituting 84.0 and 16.0% of the total effect, respectively. Social support has a direct influence on PSA and also an indirect influence through FoP. According to the Theory of Adaptation to Chronic Illness (Wang and Ye, 2021), post-surgery for primary HCC serves as a primary stimulus that affects both internal coping mechanisms (psychological resilience) and external coping mechanisms (social support) during the individual adaptation process. The individual responds to the stimulus with psychological symptoms (FoP), which manifest in varying degrees of PSA. Our findings align with theoretical models. Previous research has indicated that enhancing psychological resilience in patients with gliomas can reduce their fear of cancer recurrence (Zhong et al., 2022). Moreover, elevated levels of FoP in cancer patients lead to a cascade of psychological and social adaptation issues, including anxiety, depression, and a decline in quality of life (Dinkel et al., 2014). Adequate social support can alleviate patients’ psychological stress, facilitate better life adaptation, and thus have a direct positive impact on PSA (Al-Dwaikat et al., 2021). It can also reduce patients’ fears and anxieties regarding disease progression (Wilson et al., 2022), thereby lessening the negative impact of disease fear on psychosocial functioning (Xiong et al., 2023). These evidences indirectly support the conclusions drawn from our study. Both psychological resilience and social support have direct and indirect effects on PSA. On one hand, it emphasizes the importance of improving patients’ levels of psychological resilience and social support to enhance their PSA; on the other hand, it highlights the mediating role of FoP, suggesting that by reducing FoP levels, PSA can be improved. This provides a novel perspective on how psychological resilience and social support influence patients’ postoperative recovery.

This study also has several limitations. Firstly, our findings are derived solely from self-reported questionnaire assessments, which inherently possess subjectivity that could potentially inflate correlations between variables and introduce common method biases. Secondly, our sample originates from a single medical institution in Northwest China, thereby compromising the generalizability of our results. Thirdly, the primary variable of interest in this study, PSA, represents a dynamic process that may exhibit different trajectories over time. However, our research is cross-sectional in nature, precluding longitudinal analysis of these changes. Therefore, future research should employ longitudinal designs to explore developmental trajectories of variables and their causal relationships. Additionally, based on our study’s outcomes, multicenter intervention studies could be conducted to further investigate these aspects.

## 5 Conclusion

Postoperative patients with primary HCC exhibit lower levels of PSA. Psychological resilience and social support showed a negative correlation with FoP and a positive correlation with PSA, respectively. FoP demonstrated a negative correlation with PSA. The higher the level of psychological resilience and social support, the higher the level of PSA; The older the age, the heavier the financial burden, and the higher the level of FoP, the lower the level of PSA. FoP plays a partially mediating role between psychological resilience and PSA. Similarly, FoP partially mediates the relationship between social support and PSA.

## Data Availability

The raw data supporting the conclusions of this article will be made available by the authors, without undue reservation.
